# Overprescription of short‐acting β_2_‐agonists among patients with asthma in Saudi Arabia: Results from the SABINA III cohort study

**DOI:** 10.1111/crj.13553

**Published:** 2022-10-24

**Authors:** Hamdan Al‐Jahdali, Siraj Wali, Amr S. Albanna, Abeer Al Harbi, Riyad Allehebi, Abdulmajed Arwadi, Tarek Dahan, Mohamed Fattouh, Ezzat Hamza, Maarten Beekman

**Affiliations:** ^1^ Department of Pulmonology King Saud University for Health Sciences, College of Medicine, King Abdulaziz Medical City Riyadh Kingdom of Saudi Arabia; ^2^ King Abdullah International Medical Research Center King Saud bin Abdulaziz University for Health Sciences Jeddah Kingdom of Saudi Arabia; ^3^ Pulmonology Division King Abdulaziz University Hospital Jeddah Kingdom of Saudi Arabia; ^4^ Department of Pulmonology King Fahad General Hospital Medina Kingdom of Saudi Arabia; ^5^ Department of Pulmonology King Fahad Medical City Riyadh Kingdom of Saudi Arabia; ^6^ College of Medicine Alfaisal University Riyadh Kingdom of Saudi Arabia; ^7^ Department of Pulmonology Specialized Medical Center Hospital Riyadh Kingdom of Saudi Arabia; ^8^ Department of Pulmonology King Fahad Specialist Hospital Dammam Kingdom of Saudi Arabia; ^9^ AstraZeneca GCC Jeddah Kingdom of Saudi Arabia; ^10^ AstraZeneca The Hague The Netherlands

**Keywords:** nondrugs, prescription, asthma, bronchodilator agents, prescription, Saudi Arabia

## Abstract

Despite near‐universal health care and timely updates to treatment guidelines in Saudi Arabia, asthma control remains suboptimal, warranting deeper exploration of its management practices. This study describes asthma characteristics and prescription patterns of short‐acting β_2_‐agonists (SABAs) in the Saudi Arabia cohort of the SABA use IN Asthma (SABINA) III study. Patients with asthma (aged ≥12 years) from seven sites across Saudi Arabia participated in this cross‐sectional study. Asthma severity was classified by study investigators, guided by the 2017 Global Initiative for Asthma (GINA) recommendations. Of 511 patients enrolled, 502 patients, treated by respiratory medicine specialists, were analyzed (mean [standard deviation] age, 47.5 [14.8] years; female, 68.5%). Most patients had moderate‐to‐severe asthma (89.6%, GINA treatment steps 3–5), were overweight/obese (78.9%), and received full health care reimbursement (83.4%). Asthma was partially controlled/uncontrolled among 64.3% of patients; 62.3% experienced ≥1 severe asthma exacerbation(s), and 60.6% and 41.2% were prescribed ≥3 (overprescription) and ≥10 SABA canisters, respectively, in the 12 months preceding study initiation. Additionally, 21.9% of patients purchased SABA over the counter (OTC), of whom 66.4% purchased ≥3 SABA canisters. Ninety‐seven (88.2%) patients who purchased SABA OTC also received SABA prescriptions, and 80.4% and 56.7% of these were prescribed ≥3 and ≥10 SABA canisters, respectively. Overall, compared with SABINA III, a higher percentage of patients from Saudi Arabia were overprescribed SABA (60.6% vs. 38.0%, respectively) and purchased SABA OTC (21.9% vs. 18.0%, respectively), underscoring the need to align asthma treatment practices with current evidence‐based recommendations and regulate SABA OTC sales in Saudi Arabia.

## Graphical abstract

Results from the Saudi Arabia cohort of the SABINA III study demonstrated SABA over‐prescription (≥3 canisters/year) in more than 60% of patients with asthma, substantial over‐the‐counter purchase of SABA, and a high exacerbation burden. Our findings emphasize the need for a fundamental, paradigm shift in prescribing habits with a focus on alignment with current evidence‐based treatment recommendations.

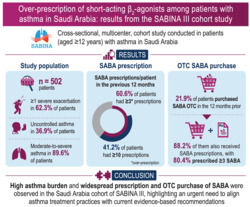

## 1 INTRODUCTION

Asthma, one of the most common, chronic respiratory diseases, is a heterogeneous syndrome characterized by chronic inflammation; variable expiratory airflow limitation; bronchial hyper‐responsiveness; and symptoms of wheezing, cough, and dyspnea, which vary in both frequency and intensity. Asthma is estimated to affect 339 million people globally and is associated with premature death and a diminished quality of life.^1^

In a 2016, population‐based, cross‐sectional study, the validated European Community Respiratory Health Survey questionnaire was administered to adult male and female residents of Riyadh, Saudi Arabia. Participants reported the prevalence of wheezing in the absence of viral rhinitis and physician‐diagnosed asthma at 18.2% and 11.3%, respectively.^2^ The high asthma prevalence in Saudi Arabia may be explained by contributing factors such as lifestyle changes; urban sprawl; dietary habits; and broad exposure to dust, tobacco smoke, sandstorms, and industrial and vehicular pollutants.^3^ Poorly controlled asthma consumes additional health care resources through increased outpatient and emergency department visits, treatment interventions, and hospital admissions for severe exacerbations. Inadequate disease control also adversely impacts the overall functional capacity, productivity, and health‐related quality of life of patients with asthma.^4^

In 2008, the Saudi Thoracic Society launched the Saudi Initiative for Asthma (SINA) group to tackle the issue of increasing incidence of asthma in the country, with the principal goal of updating management guidelines for health care practitioners who treat Saudi residents with asthma.^5^ However, despite timely revisions to the SINA guidelines and the accessibility of predominantly (60.0%) government‐funded health care facilities to Saudi nationals,^6^ sustained asthma control remains suboptimal. According to the 2018 Epidemiological Study on the Management of Asthma in Asthmatic Middle East Adult Population (ESMAA), 69.9% of patients with asthma experience partly controlled or uncontrolled disease.^7^ Recent studies on asthma treatment practices in Saudi Arabia have affirmed the high use of short‐acting β_2_‐agonist (SABA) relievers.^8^, ^9^ Moreover, long‐term SABA use as controller (maintenance) medication has been reported among 35.1% of Saudi patients with asthma.^9^ This is a cause for concern, as while SABAs provide immediate symptom relief, they do not address the underlying, chronic inflammation in asthma.^10^

Global studies have demonstrated that SABA overuse is associated with an increased risk of asthma exacerbations, incremental health care resource utilization, and even mortality.^11^, ^12^ Considering these developments, the Global Initiative for Asthma (GINA) no longer recommends SABA monotherapy. Rather, a low‐dose, inhaled corticosteroid (ICS) combined with formoterol, a long‐acting β_2_‐agonist (LABA), is now the preferred reliever for adults and adolescents at GINA treatment steps 1–2, and for patients with moderate‐to‐severe disease (treatment steps 3–5) who are prescribed ICS‐formoterol maintenance and reliever therapy.^13^ In line with GINA, the updated 2021 SINA guidelines now recommend various doses of ICS‐formoterol combination as the preferred, as‐needed reliever medication for all adults and adolescents with asthma.^14^ The SABA use IN Asthma (SABINA) III study, which is part of the global SABINA group of real‐world, observational research,^15^ was conducted to describe SABA prescription patterns in 23 countries across regions of the Middle East, Latin America, Africa, the Asia Pacific and in Russia.^16^ Here, we present results from the Saudi Arabia cohort of the SABINA III study, with the goal of better understanding patient demographics and asthma treatment practices in this country.

## 2 MATERIALS AND METHODS

### 2.1 Study design

The SABINA III study methodology has been published previously.^16^ In brief, this observational, cross‐sectional, cohort study was conducted at seven sites across Saudi Arabia, with patients recruited from Damman, Jeddah, Medina, and Riyadh between March 2019 and January 2020. A national coordinator applied purposive sampling to select study sites, with patient groups representative of current asthma management practices in the country. The principal study objective was to estimate the number of SABA and ICS prescriptions per patient in the 12 months preceding a single study visit. At each site, prespecified patient data were extracted from existing medical records. Site investigators obtained information on asthma control as well as over‐the‐counter (OTC) SABA purchases directly from the study participants and transferred those data onto electronic case report forms (eCRFs).

### 2.2 Study population

Patients aged ≥12 years with a documented diagnosis of asthma, ≥3 consultations with an HCP, medical records containing data for ≥12 months prior to study initiation, and a demonstrated ability to sign informed consent were eligible to participate in the study.

Patients with a diagnosis of other chronic respiratory diseases, such as chronic obstructive pulmonary disease, or other acute or chronic conditions, which in the opinion of the investigator would limit the ability of a patient to participate in the study, were excluded.

### 2.3 Variables and outcomes

#### 2.3.1 Prescription and purchase history

SABA prescriptions were categorized as 0, 1–2, 3–5, 6–9, 10–12, and ≥13 canisters, with overprescription defined as ≥3 SABA canisters in the 12 months prior to the study visit.^11^, ^12^ ICS prescriptions were stratified according to their respective, average, daily dose (low, medium, or high).^17^ Additionally, prescriptions for other respiratory medications, including fixed‐dose combinations of ICS with LABA, long‐term oral corticosteroids (OCS) [any treatment >10 days], OCS burst therapy (a short course of intravenous corticosteroids, or OCS administered for 3–10 days, or a single dose of an intramuscular corticosteroid to treat an exacerbation), and antibiotics prescribed for asthma, were recorded. Patients were also asked about nonprescription (OTC) SABA purchases in the 12 months preceding study initiation.

#### 2.3.2 Sociodemographic variables and disease characteristics

Other variables included sociodemographic characteristics (age, sex, number of comorbidities, body mass index [BMI], smoking status, education level [primary and/or secondary school, high school, university, and/or postgraduate]; medication reimbursement status [not reimbursed, partially reimbursed, or fully reimbursed]); practice type (primary or specialist [pulmonologist or respiratory medicine physician, general medicine physician, allergist or immunologist, or pediatrician]); asthma characteristics and treatment outcomes (investigator‐classified asthma severity [guided by the GINA 2017 treatment steps: steps 1–2, mild asthma; steps 3–5, moderate‐to‐severe asthma])^17^; and asthma duration.

#### 2.3.3 Disease outcomes

The number of severe exacerbations in the 12 months prior to the study visit, based on the American Thoracic Society/European Respiratory Society recommendations and defined as a worsening of asthma symptoms that results in hospitalization, an emergency room visit, administration of intravenous corticosteroids, an OCS prescription for three or more days, or a single dose of an intramuscular corticosteroid,^18^ was recorded in addition to asthma symptom control (based on the GINA 2017 assessment for asthma control and categorized as well controlled, partly controlled, or uncontrolled).^17^

### 2.4 Statistical analysis

All analyses were descriptive in nature. Continuous variables were summarized by the number of nonmissing values, given as mean (standard deviation [SD]) and median (range). Categorical variables were summarized by frequency counts and percentages.

## 3 RESULTS

### 3.1 Patient disposition

**FIGURE 1:**
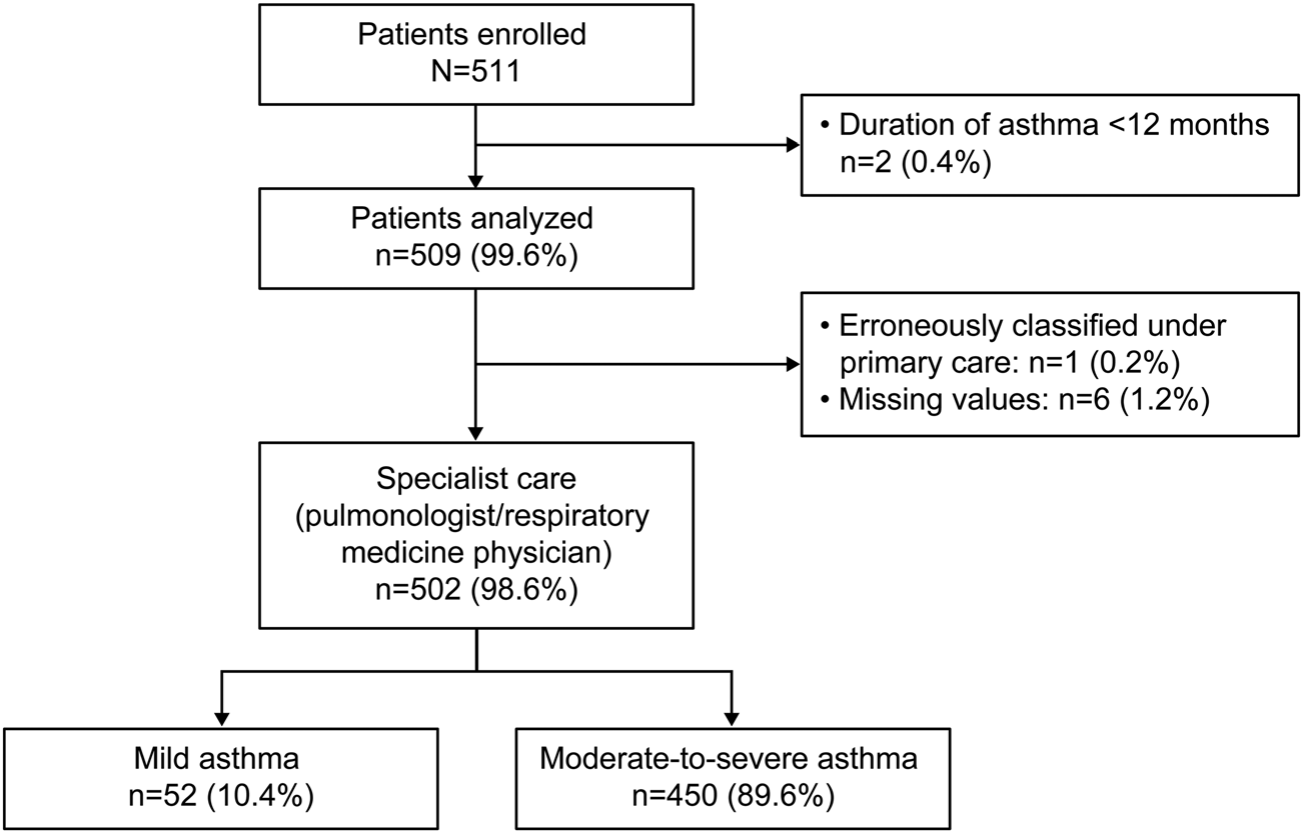
Patient disposition and study population by practice type and investigator‐classified asthma severity in the Saudi Arabia cohort of SABINA III. SABA, short‐acting β_2_‐agonist; SABINA, SABA use IN Asthma

A total of 511 patients were recruited, of whom 502 were included in the analysis. Two patients with an asthma duration of <12 months were excluded; data were missing for six patients; and one patient was likely coded erroneously as being treated by a primary care physician (PCP), rather than a specialist. Thus, all 502 analyzable patients were treated by specialists (pulmonologists or respiratory medicine physicians; Figure 1). Most patients (89.6%) were classified by investigators as having moderate‐to‐severe asthma (GINA steps 3–5), and 10.4% of patients were classified as having mild asthma (GINA steps 1–2).

### 3.2 Patient characteristics

Among the patients enrolled by respiratory medicine specialists, mean (SD) age was 47.5 (14.8) years, with approximately two thirds (65.1%) aged 18–54 years (Table 1). Patients with mild asthma were younger than those with moderate‐to‐severe disease (mean [SD] age, 40.9 [11.1] years vs. 48.3 [15.0] years). Additionally, most patients were female (68.5%) and lifelong nonsmokers (89.8%). The mean (SD) BMI of patients was 30.7 (7.3) kg/m^2^, and most patients met the World Health Organization threshold for being overweight (30.3%) or obese (48.6%). Overall, 38.3% of patients had received a university and/or post‐graduate education, while 26.8% had received a primary, secondary, or high school education. Most patients (83.4%) received fully reimbursed health care, while 16.0% were without health care reimbursement. Further, a higher proportion of patients classified with mild asthma versus moderate‐to‐severe asthma had fully reimbursed health care (96.2% vs. 82.0%). Most patients (67.5%) had ≥1 comorbidity and more patients with mild asthma had no comorbidities compared with those with moderate‐to‐severe disease (55.8% vs. 29.8%).

**TABLE 1:** Sociodemographic and disease characteristics according to investigator‐classified asthma severity and practice type in the Saudi Arabia cohort of SABINA III

Sociodemographic and disease characteristics	Specialist care		
	All (*n* = 502)	Mild asthma (*n* = 52)	Moderate‐to‐severe asthma (*n* = 450)
**Sociodemographic characteristics**			
**Age (years), mean (SD)**	47.5 (14.8)	40.9 (11.1)	48.3 (15.0)
**Age group (years)**			
12–17	7 (1.4)	1 (1.9)	6 (1.3)
18–54	327 (65.1)	44 (84.6)	283 (62.9)
≥55	168 (33.5)	7 (13.5)	161 (35.8)
**Sex (female)**	344 (68.5)	29 (55.8)	315 (70.0)
**BMI (kg/m** ^**2**^ **)**			
Mean (SD)	30.7 (7.3)	29.8 (7.1)	30.8 (7.3)
Median (min, max)	29.4 (12.8, 60.4)	28.8 (19.6, 51.4)	29.8 (12.8, 60.4)
**BMI group (kg/m** ^**2**^ **)**			
<18.5	12 (2.4)	0 (0.0)	12 (2.7)
18.5–24.9	94 (18.7)	13 (25.0)	81 (18.0)
25–29.9	152 (30.3)	19 (36.5)	133 (29.6)
≥30	244 (48.6)	20 (38.5)	224 (49.8)
**Smoking status history**			
Active smoker	28 (5.6)	5 (9.6)	23 (5.1)
Former smoker	23 (4.6)	6 (11.5)	17 (3.8)
Nonsmoker	451 (89.8)	41 (78.8)	410 (91.1)
**Number of comorbidities**			
0	163 (32.5)	29 (55.8)	134 (29.8)
≥1	339 (67.5)	23 (44.2)	316 (70.2)
**Education level**			
Primary and/or secondary school	73 (14.6)	1 (1.9)	72 (16.0)
High school	61 (12.2)	4 (7.7)	57 (12.7)
University and/or postgraduate	192 (38.3)	32 (61.5)	160 (35.6)
Unknown	175 (34.9)	15 (28.8)	160 (35.6)
Missing values	1	0	1
**Health care insurance/medication funding**			
Not reimbursed	80 (16.0)	2 (3.8)	78 (17.4)
Partially reimbursed	1 (0.2)	0 (0.0)	1 (0.2)
Fully reimbursed	418 (83.4)	50 (96.2)	368 (82.0)
Unknown	2 (0.4)	0 (0.0)	2 (0.4)
Missing values	1	0	1
**Disease characteristics**			
**Asthma duration (years)**			
Mean (SD)	14.8 (11.5)	9.5 (8.1)	15.4 (11.7)
Median (min, max)	11.0 (1.0, 77.0)	8.0 (1.0, 50.0)	11.0 (1.0, 77.0)
**Number of severe asthma exacerbations in the preceding 12 months**			
Mean (SD)	2.3 (3.7)	3.1 (5.4)	2.1 (3.5)
Median (min, max)	1.0 (0.0, 30.0)	1.0 (0.0, 30.0)	1.0 (0.0, 30.0)
Missing values	1	0	1
**Number of severe asthma exacerbations in the preceding 12 months by group**			
0	189 (37.7)	20 (38.5)	169 (37.6)
1	82 (16.4)	9 (17.3)	73 (16.3)
2	85 (17.0)	6 (11.5)	79 (17.6)
≥3	145 (29.0)	17 (32.7)	128 (28.5)
Missing values	1	0	1
**GINA classification**			
Step 1	13 (2.6)	13 (25.0)	0 (0.0)
Step 2	39 (7.8)	39 (75.0)	0 (0.0)
Step 3	73 (14.5)	0 (0.0)	73 (16.2)
Step 4	182 (36.3)	0 (0.0)	182 (40.4)
Step 5	195 (38.8)	0 (0.0)	195 (43.3)
**Level of asthma symptom control**			
Well controlled	179 (35.7)	16 (30.8)	163 (36.2)
Partly controlled	138 (27.5)	20 (38.5)	118 (26.2)
Uncontrolled	185 (36.9)	16 (30.8)	169 (37.6)

*Note*: All data are described as n (%) unless otherwise specified.

Abbreviations: BMI, body mass index; GINA, Global Initiative for Asthma; max, maximum; min, minimum; SABA, short‐acting β_2_‐agonist; SABINA, SABA use IN Asthma; SD, standard deviation.

### 3.3 Disease characteristics

Overall, mean (SD) asthma duration was 14.8 (11.5) years. Patients classified with moderate‐to‐severe and mild asthma had a mean (SD) asthma duration of 15.4 (11.7) and 9.5 (8.1) years, respectively (Table 1). The mean (SD) number of severe asthma exacerbations in the 12 months preceding the study visit was 2.3 (3.7), with 62.3% and 29.0% of patients experiencing ≥1 and ≥3 severe exacerbations, respectively. Moreover, asthma was reported as well controlled in 35.7% of the cohort, partly controlled in 27.5%, and uncontrolled in 36.9%.

### 3.4 Asthma treatment in the preceding 12 months

#### 3.4.1 SABA prescriptions

**FIGURE 2:**
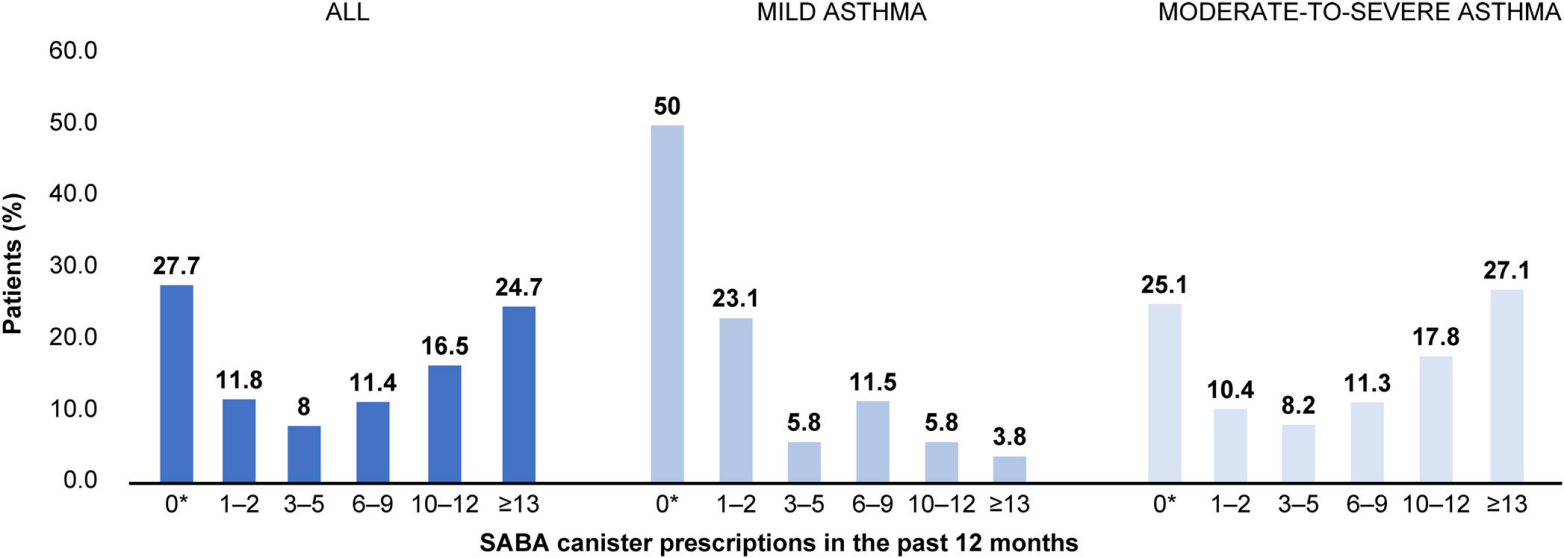
Proportion of patients receiving SABA prescriptions in the preceding 12 months according to investigator‐classified asthma severity in the Saudi Arabia cohort of SABINA III. *Patients without SABA prescriptions did not report the type of reliever they were using. SABA, short‐acting β_2_‐agonist; SABINA, SABA use IN Asthma

Overall, 60.6% of patients were prescribed ≥3 SABA canisters, with 41.2% prescribed ≥10 SABA canisters in the 12 months before study entry (Figure 2). Among patients with moderate‐to‐severe asthma, 64.4% and 44.9% were prescribed ≥3 and ≥10 SABA canisters, respectively, while 26.9% and 9.6% of those with mild disease were prescribed ≥3 and ≥10 SABA canisters, respectively.

Only 1.4% of patients, all of whom were diagnosed with mild asthma, were prescribed SABA monotherapy, with a mean (SD) of 4.3 (3.3) canisters in the previous 12 months (Table 2). However, 70.9% of patients were prescribed SABA in addition to maintenance therapy, with a mean (SD) of 11.0 (12.1) canisters in the preceding 12 months (Table 2). Of these patients, 84.3% were prescribed ≥3 canisters and 57.9% were prescribed ≥10 canisters.

**TABLE 2:** SABA prescriptions in the preceding 12 months in the Saudi Arabia cohort of SABINA III

SABA prescriptions in the preceding 12 months	Specialist care		
	All (*n* = 502)	Mild asthma (*n* = 52)	Moderate‐to‐severe asthma (*n* = 450)
**Patients who were prescribed SABA monotherapy**			
No	495 (98.6)	45 (86.5)	450 (100.0)
Yes	7 (1.4)	7 (13.5)	0 (0.0)
*Number of canisters/inhalers prescribed per patient in the preceding 12 months*			
Mean (SD)	4.3 (3.3)	4.3 (3.3)	NA
Median (min, max)	3.0 (2.0, 11.0)	3.0 (2.0, 11.0)	NA
*Number of prescriptions in the preceding 12 months (canisters/inhalers) by category*			
1–2	3 (42.9)	3 (42.9)	NA
3–5	2 (28.6)	2 (28.6)	NA
6–9	1 (14.3)	1 (14.3)	NA
10–12	1 (14.3)	1 (14.3)	NA
≥13	0 (0.0)	0 (0.0)	NA
**Patients who were prescribed SABA in addition to maintenance therapy**			
No	146 (29.1)	33 (63.5)	113 (25.1)
Yes	356 (70.9)	19 (36.5)	337 (74.9)
*Number of canisters/inhalers prescribed per patient in the preceding 12 months*			
Mean (SD)	11.0 (12.1)	5.9 (4.7)	11.3 (12.3)
Median (min, max)	11.0 (1.0, 180.0)	4.0 (2.0, 16.0)	12.0 (1.0, 180.0)
*Number of prescriptions in the preceding 12 months (canisters/inhalers) by category*			
1–2	56 (15.7)	9 (47.4)	47 (13.9)
3–5	38 (10.7)	1 (5.3)	37 (11.0)
6–9	56 (15.7)	5 (26.3)	51 (15.1)
10–12	82 (23.0)	2 (10.5)	80 (23.7)
≥13	124 (34.8)	2 (10.5)	122 (36.2)

*Note*: All data are described as n (%) unless otherwise specified.

Abbreviations: Max, maximum; min, minimum; NA, not applicable; SABA, short‐acting β_2_‐agonist; SABINA, SABA use IN Asthma; SD, standard deviation.

#### 3.4.2 OTC SABA purchase

**FIGURE 3:**
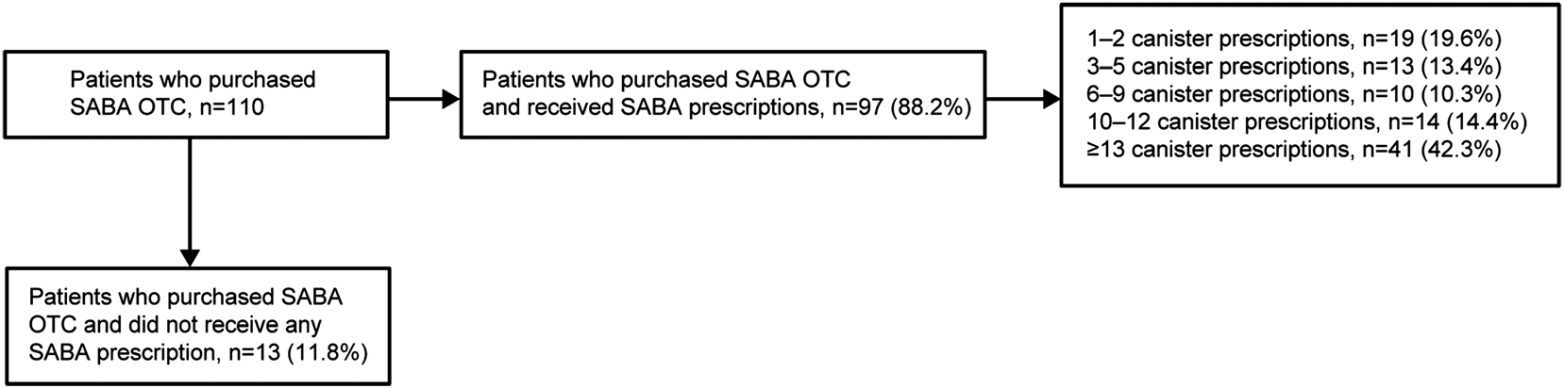
SABA purchases and prescriptions in the preceding 12 months in the Saudi Arabia cohort of SABINA III. OTC, over the counter; SABA, short‐acting β_2_‐agonist; SABINA, SABA use IN Asthma

Overall, 21.9% of patients purchased SABA OTC. Of these, 66.4% purchased ≥3 SABA canisters (Table 3). Notably, 69.3% of patients with moderate‐to‐severe asthma and 33.3% of those with mild asthma purchased ≥3 SABA canisters. Most patients who purchased SABA OTC also received SABA prescriptions (88.2%), and among these, 80.4% received ≥3 SABA prescriptions and 56.7% received ≥10 SABA prescriptions (Figure 3).

**TABLE 3:** OTC SABA purchase in the preceding 12 months in the Saudi Arabia cohort of SABINA III

OTC SABA purchase in the preceding 12 months	Specialist care		
	All (*n* = 502)	Mild asthma (*n* = 52)	Moderate‐to‐severe asthma (*n* = 450)
**Purchase of SABA without a prescription from the pharmacy in the preceding 12 months**			
No	270 (53.8)	36 (69.2)	234 (52.0)
Yes	110 (21.9)	9 (17.3)	101 (22.4)
Unknown	122 (24.3)	7 (13.5)	115 (25.6)
**Number of additional SABA in the preceding 12 months (canisters)**			
1–2	37 (33.6)	6 (66.7)	31 (30.7)
3–5	52 (47.3)	2 (22.2)	50 (49.5)
6–9	10 (9.1)	0 (0.0)	10 (9.9)
10–12	6 (5.5)	0 (0.0)	6 (5.9)
≥13	5 (4.5)	1 (11.1)	4 (4.0)

*Note*: All data are described as n (%) unless otherwise specified.

Abbreviations: OTC, over the counter; SABA, short‐acting β_2_‐agonist; SABINA, SABA use IN Asthma.

#### 3.4.3 Educational profile of patients prescribed or purchasing ≥3 SABA canisters

Among patients prescribed 0–2 and ≥3 SABA canisters, 59.3% and 30.0% had received a university and/or post‐graduate education, respectively (Table S1). Similarly, of patients who purchased 0–2 and ≥3 SABA canisters OTC, 68.7% and 39.7% reported a university and/or a postgraduate education, respectively.

### 3.5 Prescriptions for other asthma treatments

#### 3.5.1 ICS/LABA fixed‐dose combination

Most patients (96.2%) were prescribed an ICS/LABA fixed‐dose combination as maintenance therapy (Table 4), and almost all patients (99.8%) with moderate‐to‐severe disease received this combination. However, 65.4% of patients classified with mild asthma also received ICS/LABA prescriptions.

**TABLE 4:** Other asthma treatments prescribed in the preceding 12 months in the Saudi Arabia cohort of SABINA III

Asthma treatments in the preceding 12 months	Specialist care		
	All (*n* = 502)	Mild asthma (*n* = 52)	Moderate‐to‐severe asthma (*n* = 450)
**Patients prescribed ICS/LABA (fixed‐dose combinations)**			
No	19 (3.8)	18 (34.6)	1 (0.2)
Yes	483 (96.2)	34 (65.4)	449 (99.8)
*Total prescribed daily ICS dose*			
Low dose	25 (5.2)	1 (2.9)	24 (5.4)
Medium dose	262 (54.5)	26 (76.5)	236 (52.8)
High dose	194 (40.3)	7 (20.6)	187 (41.8)
Missing values	2 (0.4)	0 (0.0)	2 (0.5)
**Patients prescribed ICS**			
No	477 (95.0)	42 (80.8)	435 (96.7)
Yes	25 (5.0)	10 (19.2)	15 (3.3)
*Total prescribed daily ICS dose*			
Low dose	9 (37.5)	4 (44.4)	5 (33.3)
Medium dose	8 (33.3)	2 (22.2)	6 (40.0)
High dose	7 (29.2)	3 (33.3)	4 (26.7)
Missing values	1 (4.0)	1 (4.0)	0 (0.0)
*Total ICS prescriptions in the preceding 12 months (canisters/inhalers)*			
Mean (SD)	8.6 (6.8)	10.1 (7.2)	7.7 (6.6)
Median (min, max)	6.0 (2.0, 25.0)	9.0 (2.0, 24.0)	4.0 (2.0, 25.0)
**Patients prescribed short‐course OCS**			
No	275 (54.8)	37 (71.2)	238 (52.9)
Yes	227 (45.2)	15 (28.8)	212 (47.1)
**Patients prescribed long‐term OCS**			
No	474 (94.4)	51 (98.1)	423 (94.0)
Yes	28 (5.6)	1 (1.9)	27 (6.0)
**Patients prescribed antibiotics for asthma**			
No	406 (81.0)	49 (94.2)	357 (79.5)
Yes	95 (19.0)	3 (5.8)	92 (20.5)

*Note*: All data are described as n (%) unless otherwise specified.

Abbreviations: ICS, inhaled corticosteroid; LABA, long‐acting β_2_‐agonist; max, maximum; min, minimum; OCS, oral corticosteroid; SABA, short‐acting β_2_‐agonist; SABINA, SABA use IN Asthma; SD, standard deviation.

#### 3.5.2 Inhaled corticosteroids

Conversely, only 5.0% of patients were prescribed ICS, with a mean (SD) of 8.6 (6.8) canisters in the 12 months before study initiation (Table 4). This group comprised 3.3% of patients with moderate‐to‐severe asthma (mean [SD], 7.7 [6.6] canisters) and 19.2% with mild asthma (mean [SD], 10.1 [7.2] canisters).

#### 3.5.3 Other medications

Overall, 45.2% of patients were prescribed an OCS burst in the 12 months prior to their study visit. Further, 47.1% of patients with moderate‐to‐severe asthma and 28.8% of those with mild disease were prescribed OCS (Table 4).

A total of 19.0% of patients were prescribed an antibiotic. Antibiotics were prescribed more commonly to patients with moderate‐to‐severe disease than to those with mild asthma (20.5% vs. 5.8% [Table 4]).

Additionally, 20.5% of patients were prescribed biologic agents (data not shown). Of these, 67.0% were prescribed omalizumab, 27.2%, mepolizumab, 2.9%, a combination of omalizumab with mepolizumab, and 2.9% received dupilumab.

### 3.6 Asthma treatments and exacerbations

**FIGURE 4:**
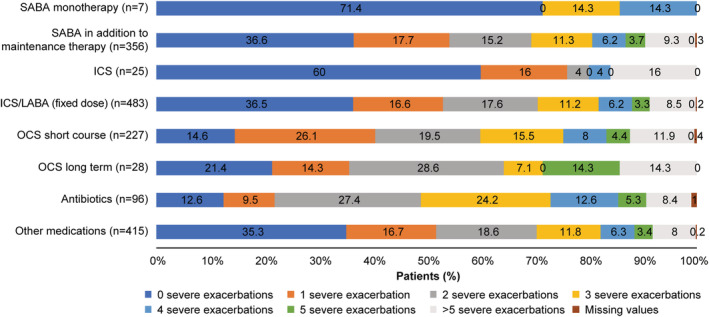
Severe asthma exacerbations and treatments in the preceding 12 months in the Saudi Arabia cohort of SABINA III. ICS, inhaled corticosteroid; LABA, long‐acting β_2_‐agonist; OCS, oral corticosteroids; SABA, short‐acting β_2_‐agonist; SABINA, SABA use IN Asthma

When stratified by treatments prescribed in the 12 months preceding their study visit, most patients prescribed antibiotics (87.4%) experienced ≥1 severe exacerbation, followed by those prescribed short‐term OCS (85.4%), long‐term OCS (78.6%), ICS/LABA fixed‐dose combinations (63.5%), SABA in addition to maintenance therapy (63.4%), ICS (40.0%), and SABA monotherapy (28.6%; Figure 4).

## 4 DISCUSSION

Overall, results from this cross‐sectional, observational, cohort study conducted at seven sites across Saudi Arabia demonstrated a high percentage of SABA overprescription in the country, suggesting substantial overreliance on SABA for rapid symptomatic relief. Our findings align with those from previous reports on asthma management practices in Saudi Arabia, which emphasized a strong preference for SABA therapy.^8^, ^9^ A 2018 cross‐sectional study, which assessed the utilization patterns of asthma medications at primary health care centers in Saudi Arabia, reported long‐term use of SABA as a controller agent by approximately one third of patients,^9^ suggesting a history of improper use. Because inappropriate SABA use has been linked to adverse consequences^11^, ^12^ and country‐aggregated data from the SABINA Pan International study demonstrated a relationship between SABA prescription volume and poor clinical outcomes,^16^ improving asthma treatment practices in Saudi Arabia becomes critical. This undertaking will require wide‐ranging modifications in prescribing habits, patient behavior, and health care policy at the national level.

Of concern, 60.6% of patients from Saudi Arabia were overprescribed SABA. Although these prescription patterns were comparable to those reported in the Gulf cohort (Kuwait, Oman, and the United Arab Emirates) of the SABINA III study where 58.5% patients were overprescribed SABA,^19^ this was considerably higher than that observed in the overall SABINA III cohort of 8351 patients where 38.0% of patients received ≥3 SABA prescriptions,^16^ and the Middle Eastern (*n* = 1389),^20^ African (*n* = 1778),^21^ Latin American (*n* = 1096),^22^ and Asian (*n* = 3066)^23^ cohorts of SABINA III, where 47.1%, 46.5%, 39.8%, and 26.1% of patients, respectively, were prescribed ≥3 SABA canisters in the 12 months prior to study initiation. Similarly, rates of SABA overprescription in this Saudi Arabian cohort were higher than those reported from a pan‐European study, which was conducted as part of the SABINA programme in 1.06 million patients with asthma, and reported the prevalence of SABA overuse to be 9.0% in Italy, 16.0% in Germany, 29.0% in Spain, 30.0% in Sweden, and 38.0% in the United Kingdom.^24^ Moreover, results from observational analyses of 10 SABINA datasets involving 1 033 564 patients from Canada, France, the Netherlands, Poland, Spain, the United Kingdom and the United States reported that 40.2% of patients were prescribed/possessed ≥3 SABA canisters/year,^25^ which again was lower than that reported in this cohort of patients from Saudi Arabia. Unsurprisingly, therefore, the burden of disease appeared to be greater in Saudi Arabia, given that the proportion of patients with ≥1 severe asthma exacerbation(s) in the 12 months preceding their study visit was higher than that observed in the total SABINA III population (62.3% vs. 45.4%).^16^ The percentage of patients with uncontrolled asthma also was higher in the SABINA Saudi Arabia cohort (36.9%) compared with that in the SABINA III study (24.5%).^16^ Interestingly, the Asthma Insights and Reality in the Gulf and the Near East (AIRGNE) survey that reported SABA use in 55.5% of patients with a low ICS:SABA ratio across Jordan, Kuwait, Lebanon, Oman, and the United Arab Emirates, cited overestimation of asthma control, underuse of appropriate controller treatment, and lack of patient education as some of the factors responsible for SABA overreliance in the region.^26^ Indeed, in our study, the percentage of patients with university/post‐graduate education was considerably higher among patients prescribed 0–2 SABA canisters (59.3%) compared with those overprescribed SABA (30.0%). This suggests a potential correlation of higher educational status with lower SABA prescription; however, these observations must be interpreted with caution because the educational status of approximately 35.0% of the cohort was unknown. Further, asthma control in Saudi Arabia was similar to that observed in other countries in the Middle East, with one study on adult asthma in the Middle East and North Africa reporting uncontrolled disease in 41.5% of patients,^27^ a finding supported by a Gulf region study showing a 39.2% prevalence of uncontrolled asthma.^28^

Despite the observation that most participants in this study benefited from a medication reimbursement plan, a high proportion of those who purchased SABA OTC obtained ≥3 SABA canisters in the 12 months prior to their study visit. Of additional concern, most patients who purchased SABA OTC also received SABA prescriptions, indicating possession and possible use of an unwarranted quantity by such patients. This behavior may be ascribed to the easy access and ready availability of asthma medication in Saudi Arabia without guideline‐directed evidence for its indication.^29^ Thus, our findings make a compelling case for health system improvements at a national policy level to better regulate SABA OTC purchase and discourage over prescription of SABA monotherapy in Saudi Arabia. Additionally, as pharmacists occupy the front line in dispensing OTC medications, organizing awareness programmes to familiarize retail pharmacists with updated treatment guidelines could mitigate SABA over prescription and noncompliance among patients who purchase it OTC.

Of interest, 29.1% of patients in the Saudi Arabia cohort did not receive a prescription for SABA in addition to their maintenance medication, suggesting that alternative, rapid‐onset relievers recommended by SINA, such as a combination of budesonide and formoterol^30^ or ipratropium bromide,^30^ might have been prescribed for immediate symptom control. Further, while 62.3% of patients experienced ≥1 severe asthma exacerbation(s) in the 12 months preceding study initiation, only 45.2% were prescribed OCS burst therapy. Given that asthma exacerbations are managed primarily with this regimen,^31^ it is possible that patients purchased OCS without a prescription to treat asthma exacerbations or received a short course of OCS during emergency room visits, data on which were not captured in this study. Alternatively, given the alarming number of antibiotic prescriptions for asthma (19.0%) recorded in the study, antibiotics may also have been prescribed for the treatment of asthma exacerbations in patients with moderate‐to‐severe disease. However, this may not align with SINA guidelines,^30^ which recommend that antibiotics be restricted to cases with strong evidence of bacterial pneumonia in patients with severe asthma who do not qualify for or respond to biologic therapy. Across all SABINA III countries,^16^ the Saudi Arabia cohort accounted for 39.0% of all study participants prescribed biologic agents for the treatment of asthma and approximately 20.0% of patients were prescribed biologics, most commonly with omalizumab.

The high clinical and socio‐economic burden of asthma and widespread SABA over prescription in Saudi Arabia may exist due to several barriers to optimal asthma care reported across different studies. These include patient‐related factors such as lack of education on the disease state and its corresponding levels of severity, guideline‐directed asthma treatment steps,^32^ improper inhaler technique,^33^ suboptimal clinical care, and poor adherence to individualized action plans^34^; physician‐related factors, such as insufficient knowledge and implementation of asthma management guidelines^34^; and societal factors, including the tendency to self‐medicate via OTC purchases of prescription drugs,^29^ and misinformation on proper inhaler technique from pharmacists who dispense asthma medication.^35^ Thus, while Saudi Arabia recently updated its guidelines^14^ to align with those of GINA treatment strategies, such that SABA is no longer the preferred rapid‐onset reliever, an unmet need prevails to launch educational initiatives that target physicians, pharmacists, and patients to ensure that these updated guidelines are adopted in clinical practice. This is especially important in Saudi Arabia because our findings indicate that despite a large fraction of patients having received a university and/or post‐graduate education, they were overprescribed SABA and/or had purchased ≥3 SABA canisters OTC. Thus, in addition to increasing awareness of guideline‐directed disease management among clinicians, patient‐directed asthma education in Saudi Arabia also warrants urgent prioritization. In line with this, the Ministry of Health in Saudi Arabia launched an asthma pocket guide in 2020,^36^ which is intended to serve as a quick and accessible reference for health care professionals and patients and includes a step‐by‐step action plan for asthma treatment and control guided by the GINA 2020 report.^37^ While this is a step in the right direction, more educational campaigns and community outreach initiatives are needed to raise awareness among patients with asthma and their health care practitioners on suitable and accessible GINA treatment step options.

Our study clearly demonstrated a high asthma burden in Saudi Arabia; however, it is not without its limitations. All patients in this study were treated by pulmonologists or other respiratory specialists, and as a result, primary care was not represented. Consequently, our observations potentially may underestimate the countrywide burden of disease and not reflect the full spectrum of asthma care practices in Saudi Arabia. In accordance with this, a cross‐sectional study examining asthma medication patterns in primary care reported SABA use in 90.3% of patients,^9^ suggesting a potentially higher prevalence of SABA over prescription in Saudi Arabia than what has been captured in this study. Our study also did not record information on the use of alternative relievers such as ICS‐formoterol combinations. Further, prescription data may not always reflect actual medication use, and rates of treatment compliance or noncompliance were unknown. Additionally, data extraction from patient medical records relied on clinician assessment and accurate transcription, and while unlikely, our findings may have been impacted by misinterpretation of instructions, incorrect patient classification, or recall bias in the case of SABA OTC purchases. Finally, as the study primarily focused on the volume of SABA prescriptions, the potential overuse of oral (tablets) or nebulized dosage forms of SABA was not captured. Despite these limitations, our data on the substantial burden of SABA over prescription across Saudi Arabia heightens the urgency to improve asthma management practices and serves as a call to action for clinicians and policymakers alike to ensure nationwide adoption of contemporary treatment guidelines with the ultimate goal of optimizing and sustaining disease control among patients with asthma. It is likely that similar trends may be observed in primary care settings, and future real‐world studies of prescription data may further assist in improving our understanding of asthma care among frontline providers.

In conclusion, SABA overprescription (≥3 canisters per year) was widespread, occurring in two thirds of patients in Saudi Arabia. This was considerably higher than that reported in the overall SABINA III population,^16^ where just over one third of patients reported overprescription of SABAs. In addition, unregulated access to SABA was common, with nearly a quarter of patients purchasing SABA OTC. These findings highlight that SABA overprescription is a major public health concern in Saudi Arabia, emphasizing the need for a fundamental, paradigm shift in prescribing habits and alignment of clinical practices with the latest evidence‐based recommendations to improve and sustain disease control and reduce asthma exacerbation rates.

## CONFLICT OF INTEREST

HA‐J, SW, ASA, AAH, RA, AA, and TD have no conflict of interest to declare. MF and EZ are employees of AstraZeneca. MB was an employee of AstraZeneca at the time the study was conducted.

## ETHICS STATEMENT

The study was conducted in compliance with the study protocol and Declaration of Helsinki. Local ethics approval was obtained from the King Abdullah International Medical Center Institutional Review Board.

## AUTHOR CONTRIBUTIONS

MB designed the study. HA‐J, SW, ASA, AAH, RA, AA, and TD contributed to data collection. All authors contributed to data analysis, data interpretation and drafting and reviewing the manuscript.

## Supporting information


**Table S1.** SABA prescription/purchase and education level in the Saudi Arabia cohort of SABINA III

## Data Availability

Data underlying the findings described in this manuscript may be obtained in accordance with AstraZeneca's data sharing policy described at https://astrazenecagrouptrials.pharmacm.com/ST/Submission/Disclosure.

## References

[1] Executive Summary. The Global Asthma Report 2018. Global Asthma Report. Accessed April 24, 2021. http://globalasthmareport.org/foreword/summaries.php

[2] Al Ghobain MO , Algazlan SS , Oreibi TM . Asthma prevalence among adults in Saudi Arabia. Saudi Med J. 2018;39(2):179‐184. doi:10.15537/smj.2018.2.20974 29436567 PMC5885095

[3] Mohamed Hussain S , Ayesha Farhana S , Mohammed Alnasser S . Time trends and regional variation in prevalence of asthma and associated factors in Saudi Arabia: a systematic review and meta‐analysis. Biomed Res Int. 2018;2018:8102527. doi:10.1155/2018/8102527 29951546 PMC5989288

[4] Gold LS , Thompson P , Salvi S , Faruqi RA , Sullivan SD . Level of asthma control and health care utilization in Asia‐Pacific countries. Respir Med. 2014;108(2):271‐277. doi:10.1016/j.rmed.2013.12.004 24406243

[5] al‐Moamary MS , al‐Hajjaj MS , Idrees M , et al. The Saudi initiative for asthma. Ann Thorac Med. 2009;4(4):216‐233. doi:10.4103/1817-1737.56001 19881170 PMC2801049

[6] al Asmri M , Almalki MJ , Fitzgerald G , Clark M . The public health care system and primary care services in Saudi Arabia: a system in transition. East Mediterr Health J. 2020;26(4):468‐476. doi:10.26719/emhj.19.049 32338366

[7] Al‐Jahdali H , Wali S , Salem G , et al. Asthma control and predictive factors among adults in Saudi Arabia: results from the epidemiological study on the management of Asthma in asthmatic Middle East adult population study. Ann Thorac Med. 2019;14(2):148‐154. doi:10.4103/atm.ATM_348_18 31007767 PMC6467022

[8] Rafeeq M , Murad H . Evaluation of drug utilization pattern for patients of bronchial asthma in a government hospital of Saudi Arabia. Niger J Clin Pract. 2017;20(9):1098‐1105. doi:10.4103/njcp.njcp_378_16 29072231

[9] Alqahtani NS , Almardhamah NH , Almaqbul WN , et al. The pattern of use of bronchial asthma medications at primary health care level and the factors influencing this from the physicians' perspective, Najran city, Saudi Arabia. IJMDC. 2019;3(8):686‐693.

[10] Aldridge RE , Hancox RJ , Robin Taylor D , et al. Effects of terbutaline and budesonide on sputum cells and bronchial hyperresponsiveness in asthma. Am J Respir Crit Care Med. 2000;161(5):1459‐1464. doi:10.1164/ajrccm.161.5.9906052 10806139

[11] Bloom CI , Cabrera C , Arnetorp S , et al. Asthma‐related health outcomes associated with short‐acting β_2_‐agonist inhaler use: an observational UK study as part of the SABINA global program. Adv Ther. 2020;37(10):4190‐4208. doi:10.1007/s12325-020-01444-5 32720299

[12] Nwaru BI , Ekström M , Hasvold P , Wiklund F , Telg G , Janson C . Overuse of short‐acting β_2_‐agonists in asthma is associated with increased risk of exacerbation and mortality: a nationwide cohort study of the global SABINA programme. Eur Respir J. 2020;55(4):1901872. doi:10.1183/13993003.01872-2019 31949111 PMC7160635

[13] Global strategy for asthma management and prevention. Global Initiative for Asthma (GINA) Updated 2022. Accessed August 29, 2022. https://ginasthma.org/wp-content/uploads/2022/07/GINA-Main-Report-2022-FINAL-22-07-01-WMS.pdf

[14] al‐Moamary MS , Alhaider SA , Alangari AA , et al. The Saudi Initiative for Asthma—2021 update: guidelines for the diagnosis and management of asthma in adults and children. Ann Thorac Med. 2021;16(1):4‐56. doi:10.4103/atm.ATM_697_20 33680125 PMC7908897

[15] Cabrera CS , Nan C , Lindarck N , Beekman M , Arnetorp S , van der Valk RJP . SABINA: global programme to evaluate prescriptions and clinical outcomes related to short‐acting β_2_‐agonist use in asthma. Eur Respir J. 2020;55(2):1901858. doi:10.1183/13993003.01858-2019 31806716

[16] Bateman ED , Price DB , Wang HC , et al. Short‐acting β_2_‐agonist prescriptions are associated with poor clinical outcomes of asthma: the multi‐country, cross‐sectional SABINA III study. Eur Respir J. 2022;59(5):2101402. doi:10.1183/13993003.01402-2021 34561293 PMC9068976

[17] Global strategy for asthma management and prevention. Global Initiative for Asthma (GINA) 2017. Accessed April 25, 2021. https://ginasthma.org/wp-content/uploads/2019/01/2017-GINA.pdf

[18] Reddel HK , Taylor DR , Bateman ED , et al. An official American Thoracic Society/European Respiratory Society statement: asthma control and exacerbations: standardizing endpoints for clinical asthma trials and clinical practice. Am J Respir Crit Care Med. 2009;180(1):59‐99. doi:10.1164/rccm.200801-060ST 19535666

[19] Alzaabi A , al Busaidi N , Pradhan R , et al. Over‐prescription of short‐acting β_2_‐agonists and asthma management in the Gulf region: a multicountry observational study. Asthma Res Pract. 2022;8(1):3. doi:10.1186/s40733-022-00085-5 35799290 PMC9260980

[20] Al Zaabi A , Busaidi N , Al Mutairy S , et al. Overprescription of short‐acting β_2_‐agonists is associated with poor asthma symptom control: results from five Middle Eastern countries included in the SABINA International (III) study. Expert Rev Respir Med. 2022;16(7):833‐847. doi:10.1080/17476348.2022.2099841 35848074

[21] Khattab A , Madkour A , Ambaram A , et al. Over‐prescription of short‐acting β_2_‐agonists is associated with poor asthma outcomes: results from the African cohort of the SABINA III study. Curr Med Res Opin. 2022;1‐13. doi:10.1080/03007995.2022.2100649 36031882

[22] Montero‐Arias F , Garcia JCH , Gallego MP , et al. Over‐prescription of short‐acting β_2_‐agonists is associated with poor asthma outcomes: results from the Latin American cohort of the SABINA III study. J Asthma. 2022;1‐14. doi:10.1080/02770903.2022.2082305 35670783

[23] Wang H‐C , Djajalaksana S , Sharma L , et al. 2021. P9‐23: Prevalence of SABA over‐prescription and association with clinical outcomes for asthma management in primary care in the Asian cohort of SABINA III. Paper presented at: The 25th Annual Congress of the Asian Pacific Society of Respirology; November 20–21, 2021; Kyoto, Japan.

[24] Janson C , Menzies‐Gow A , Nan C , et al. SABINA: an overview of short‐acting β_2_‐agonist use in asthma in European countries. Adv Ther. 2020;37(3):1124‐1135. doi:10.1007/s12325-020-01233-0 31981105 PMC7089727

[25] Quint JK , Arnetorp S , Kocks JWH , et al. Short‐acting β_2_‐agonist exposure and severe asthma exacerbations: SABINA findings from Europe and North America. J Allergy Clin Immunol Pract. 2022;10(9):2297‐2309. doi:10.1016/j.jaip.2022.02.047 35364341

[26] Khadadah M , Mahboub B , Al‐Busaidi NH , Sliman N , Soriano JB , Bahous J . Asthma insights and reality in the Gulf and the near East. Int J Tuberc Lung Dis. 2009;13(8):1015‐1022. 19723383

[27] Tarraf H , Al‐Jahdali H , Al Qaseer AH , et al. Asthma control in adults in the Middle East and North Africa: results from the ESMAA study. Respir Med. 2018;138:64‐73. doi:10.1016/j.rmed.2018.03.024 29724395

[28] Alzaabi A , Idrees M , Behbehani N , Salah F . Patients' and physicians' attitudes and perception about asthma in the Gulf: a subset analysis from the Asthma Insights and Management Survey in the Gulf and Russia. Allergy Asthma Proc. 2021;42(3):e77‐e85. doi:10.2500/aap.2021.42.210027 33980343

[29] Ijpnl C , Al‐mutairi AM , Al‐enezi DH , et al. Asthma inhalers sale without medical prescription in Riyadh, Saudi Arabia: a cross sectional study. Int J Pharm. 2014;4(2):24‐27.

[30] Al‐Moamary MS , Alhaider SA , Alangari AA , et al. The Saudi Initiative for Asthma—2019 update: guidelines for the diagnosis and management of asthma in adults and children. Ann Thorac Med. 2019;14(1):3‐48. doi:10.4103/atm.ATM_327_18 30745934 PMC6341863

[31] Fuhlbrigge AL , Lemanske RF Jr , Rasouliyan L , Sorkness CA , Fish JE . Practice patterns for oral corticosteroid burst therapy in the outpatient management of acute asthma exacerbations. Allergy Asthma Proc. 2012;33(1):82‐89. doi:10.2500/aap.2012.33.3499 22183118

[32] Noibi S , Mohy A , Gouhar R , Shaker F , Lukic T , Al‐Jahdali H . Asthma control factors in the Gulf Cooperation Council (GCC) countries and the effectiveness of ICS/LABA fixed dose combinations: a dual rapid literature review. BMC Public Health. 2020;20(1):1211. doi:10.1186/s12889-020-09259-3 32770967 PMC7414753

[33] Al‐Jahdali H , Ahmed A , Al‐Harbi A , et al. Improper inhaler technique is associated with poor asthma control and frequent emergency department visits. Allergy Asthma Clin Immunol. 2013;9(1):8. doi:10.1186/1710-1492-9-8 23510684 PMC3605255

[34] Braido F . Failure in asthma control: reasons and consequences. Scientifica (Cairo). 2013;2013:549252. doi:10.1155/2013/549252 24455432 PMC3881662

[35] Khan TM , Azhar S . A study investigating the community pharmacist knowledge about the appropriate use of inhaler, Eastern Region AlAhsa, Saudi Arabia. Saudi Pharm J. 2013;21(2):153‐157. doi:10.1016/j.jsps.2012.07.004 23960829 PMC3744930

[36] Asthma Pocket Guide for Health Care Professionals. Ministry of Health. 2020. Accessed June 16, 2021. https://www.moh.gov.sa/en/Ministry/About/Health%20Policies/Asthma-Pocket-Guide-for-Health-Care-Professionals.pdf

[37] Global strategy for asthma management and prevention. Global Initiative for Asthma (GINA). 2020. Accessed April 25, 2021. https://ginasthma.org/wp-content/uploads/2020/06/GINA-2020-report_20_06_04-1-wms.pdf

