# Supplementary material for: Overprescription of short‐acting β2‐agonists among patients with asthma in Saudi Arabia: Results from the SABINA III cohort study
Source: Clin Respir J. 2022 Oct 24;16(12):812–25. doi: 10.1111/crj.13553 (PMC9716708; doi:10.1111/crj.13553)
Supplement: Supplementary file 1 — Table S1. SABA prescription/purchase and education level in the Saudi Arabia cohort of SABINA III [file CRJ-16-812-s001.docx]

**SUPPORTING INFORMATION**

**Table S1** SABA prescription/purchase and education level in the Saudi Arabia cohort of SABINA III

| **Education level** | **All** | **0–2 SABA canisters** | **≥3 SABA canisters** |
| --- | --- | --- | --- |
| **SABA prescriptions** | | | |
| Primary and/or secondary school | 45 (12.4) | 6 (10.2) | 39 (12.9) |
| High school | 58 (16.0) | 5 (8.5) | 53 (17.5) |
| University and/or postgraduate | 126 (34.8) | 35 (59.3) | 91 (30.0) |
| Unknown | 133 (36.7) | 13 (22.0) | 120 (39.6) |
| Missing values | 1 | 0 | 1 |
| Total | 363 | 59 | 304 |
| **SABA purchase** | | | |
| Primary and/or secondary school | 19 (17.3) | 4 (12.5) | 15 (19.2) |
| High school | 19 (17.3) | 2 (6.3) | 17 (21.8) |
| University and/or postgraduate | 53 (48.2) | 22 (68.7) | 31 (39.7) |
| Unknown | 19 (17.3) | 4 (12.5) | 15 (19.2) |
| Missing values | 0 | 0 | 0 |
| Total | 110 | 32 | 78 |

All data are described as n (%) unless otherwise specified.

SABA, short‑acting β_2_-agonist; SABINA, SABA use IN Asthma.
